# RETRACTED: Elsherif et al. Investigation of the Potential of Nebivolol Hydrochloride-Loaded Chitosomal Systems for Tissue Regeneration: In Vitro Characterization and In Vivo Assessment. *Pharmaceutics* 2021, *13*, 700

**DOI:** 10.3390/pharmaceutics17010038

**Published:** 2024-12-30

**Authors:** Noha Ibrahim Elsherif, Abdulaziz Mohsen Al-Mahallawi, Abdelfattah Ahmed Abdelkhalek, Rehab Nabil Shamma

**Affiliations:** 1 Department of Pharmaceutics and Pharmaceutical Technology, Faculty of Pharmacy, Heliopolis University, Cairo 11785, Egypt; 2 Department of Pharmaceutics and Industrial Pharmacy, Faculty of Pharmacy, Cairo University, Cairo 11562, Egypt; 3 Department of Pharmaceutics, Faculty of Pharmacy, October University for Modern Sciences and Arts (MSA), Giza 12451, Egypt; 4 Department of Microbiology of Supplementary General Science, Faculty of Oral and Dental Medicine, Future University in Egypt, Cairo 11835, Egypt

The journal retracts the article “Investigation of the Potential of Nebivolol Hydrochloride-Loaded Chitosomal Systems for Tissue Regeneration: In Vitro Characterization and In Vivo Assessment” [1], cited above.

Following publication, concerns were brought to the attention of the publisher regarding the presence of image irregularities with a figure presented within this publication [1].

Adhering to our complaints procedure, an investigation was conducted by the Editorial Office and Editorial Board, which confirmed a range of inappropriate image modifications to panels presented in Figure 6. While the authors fully cooperated with the Editorial Office during the investigation, they were unable to satisfactorily explain the above-mentioned concerns, nor provide appropriate raw material for Editorial Board evaluation. As a result, the Editorial Board has lost confidence in the integrity of the findings and decided to retract this publication [1], as per MDPI’s retraction policy (https://www.mdpi.com/ethics#_bookmark30).

This retraction was approved by the Editor-in-Chief of the journal *Pharmaceutics*.

The authors agree to this retraction.
